# Synthesis and Antimalarial Activity of 1,4-Disubstituted Piperidine Derivatives

**DOI:** 10.3390/molecules25020299

**Published:** 2020-01-11

**Authors:** Rokhyatou Seck, Abdoulaye Gassama, Sandrine Cojean, Christian Cavé

**Affiliations:** 1 Laboratoire de Chimie et Physique des Matériaux (LCPM), Université Assane SECK de Ziguinchor, Ziguinchor BP 523, Senegal; seckrokhya@gmail.com; 2 Centre National de Référence du Paludisme, Hôpital Bichat-Claude Bernard, APHP, 75018 Paris, France; sandrine.cojean@u-psud.fr; 3 Université Paris-Saclay, CNRS BioCIS, 92290 Châtenay-Malabry, France

**Keywords:** piperidine, reductive amination, reagent-based diversity, antimalarial, drug lead

## Abstract

In order to prepare, at low cost, new compounds active against *Plasmodium falciparum*, and with a less side-effects, we have designed and synthesized a library of 1,4-disubstituted piperidine derivatives from 4-aminopiperidine derivatives **6**. The resulting compound library has been evaluated against chloroquine-sensitive (3D7) and chloroquine-resistant (W2) strains of *P. falciparum.* The most active molecules—compounds **12d** (13.64 nM (3D7)), **13b** (4.19 nM (3D7) and 13.30 nM (W2)), and **12a** (11.6 nM (W2))—were comparable to chloroquine (22.38 nM (3D7) and 134.12 nM (W2)).

## 1. Introduction

This year’s World Health Organization (WHO) report shows that after an unprecedented period of success in global malaria control, progress has stalled [1]. In 2016, there were an estimated 216 million cases of malaria, an increase of about 5 million cases over 2015. Deaths reached 445,000, a similar number to the previous year.

Malaria-related mortality followed the same trend, i.e., a decline from 2010 to 2014, and then an increase in 2015 and 2016. According to this report, it is in the WHO African region that the increase in cases of malaria and associated deaths was the most significant. The African region still accounts for some 90% of worldwide malaria cases and related deaths. Fifteen countries, all but one in sub-Saharan Africa, account for 80% of the global burden of malaria.

One of the biggest challenges facing malaria chemotherapy is the rapid emergence of resistance to existing antimalarial drugs [2]. Chloroquine was replaced as first line therapy by the sulfonamide antimalarials and, later on, artemisinin combination therapy (ACT), following the development of widespread resistance against the drug by *Plasmodium falciparum* [3]. This challenge underscores the need for the continued search for new antimalarials.

The 4-arylaminopiperidine is a structural moiety found in many alkaloids [4,5,6,7,8,9,10,11] and pharmaceutical products such as fentanyl and structurally-related analgesic opioids or H1-antihistamines agents such as bamipine [12,13,14,15,16,17] and neurokinin 1 (NK1) receptor antagonists [18,19,20]. Studies have shown that compounds with piperidine rings [4,8,21,22,23,24,25,26] have good selectivity and activity for the *P. falciparum* strain.

**Figure 1:**
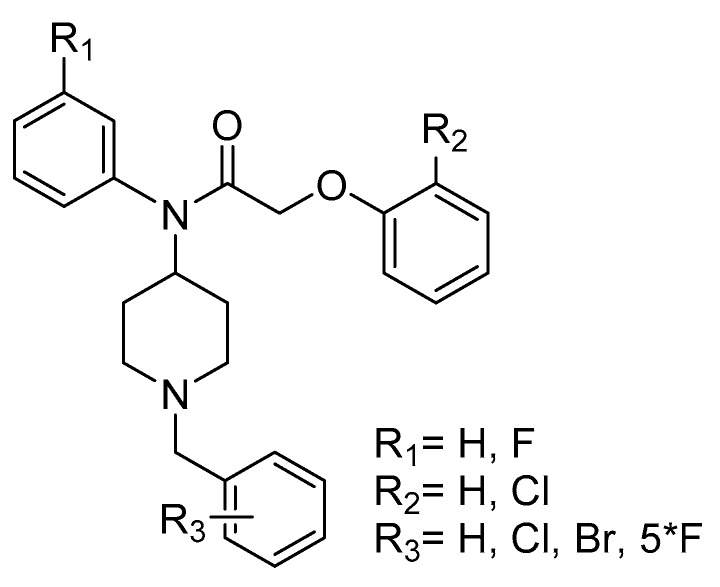
target compounds **A**.

Research is being pursued for the discovery of new antimalarials with less side effects, a faster onset of action and a better rate of response [4,8,21,22,23,24,25,26]. In the process of searching for new small molecules interacting with the *P. falciparum* strain, we have identified the target compounds **A** with various R1, R2 and R3 substituents (Figure 1). In this paper, we describe the synthesis of some new derivatives with potential antimalarial properties.

## 2. Results and Discussion

### 2.1. Chemistry

**Scheme 1:**
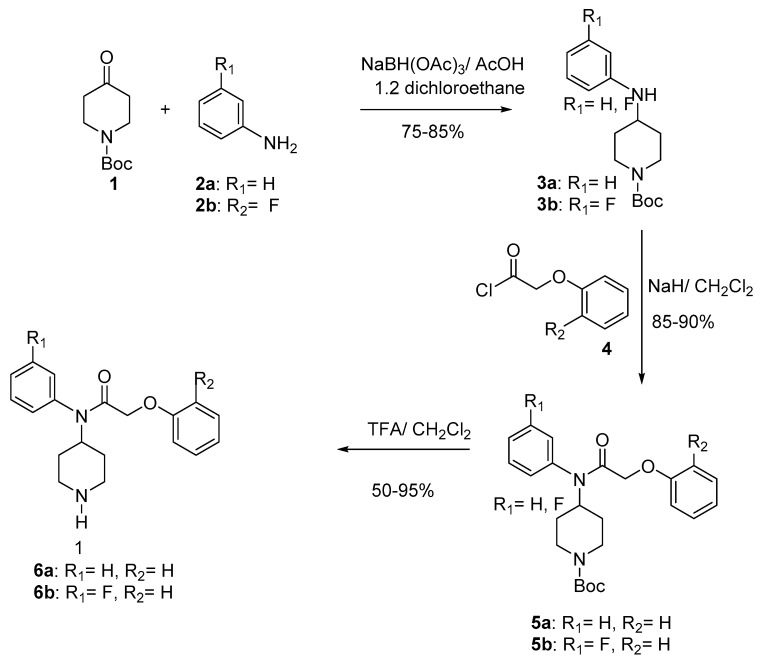
Synthesis of compounds **6a**–**b.**

The key compound **6** has been synthesized through a three-step process according to the Scheme 1 [27]. Thus, reductive amination [28,29,30,31] of *N*-boc-piperidin-4-one (**1**) with anilines **2a, b** in CH_2_Cl_2_, gave compounds **3a, b** in 75–85% yield. Acylation of the sodium salts of **3** with phenoxyacetyl chlorides **4** in CH_2_Cl_2_ at 0 °C furnished compounds **5** (85–90%). Final deprotection [32,33] of **5** using trifluoroacetic acid at room temperature provided compounds **6a–b** (50–95%) (Scheme 1).

**Scheme 2:**
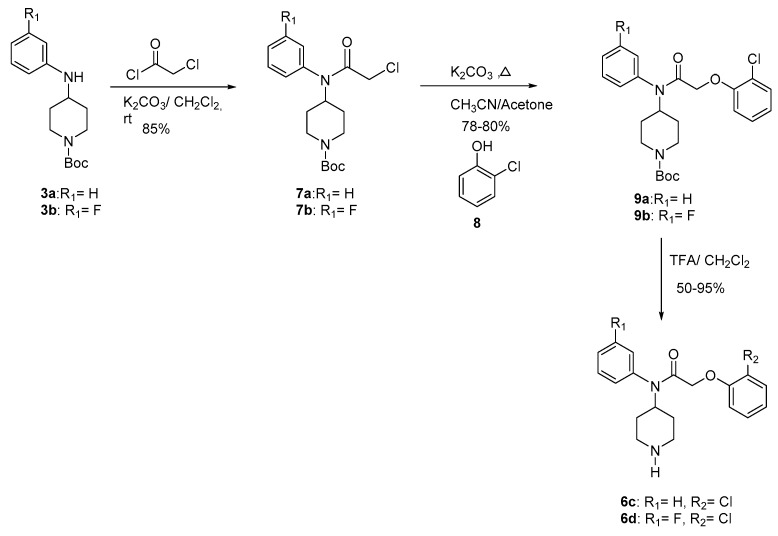
Synthesis of compound **6c** and **6d.**

The alkylation of **3** with the acetyl chloride supplied compounds **7** in 85% yield. The condensation of **7** with the phenol **8** gave compounds **9** (78–80%). Final deprotection [32,33] of **9** using trifluoroacetic acid at room temperature provided compounds **6c**–**d** (50–95%) (Scheme 2). The Table 1 gives the overall yields of compounds **6a**–**d.**

**Scheme 3:**
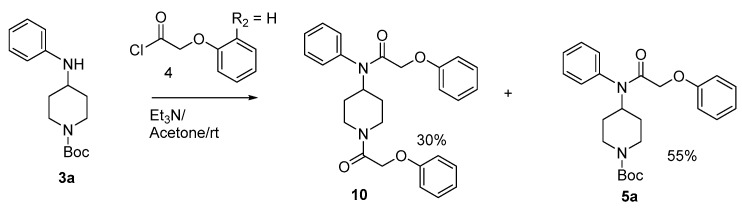
Synthesis of compounds **5a** and **10.**

Condensation of phenoxyacetyl chloride with compound **3a** in the presence of triethylamine at room temperature in acetone gave compounds **5a** (55%) and **10** (30%) (Scheme 3). In this reaction we used excess phenoxyacetyl chloride, and we think that this excess probably made the reaction medium acidic which cause the cleavage of the N-Boc protective group. To avoid this side reaction, we used NaH in CH_2_Cl_2_ which furnished compound **5a** (Scheme 1).

**Scheme 4:**
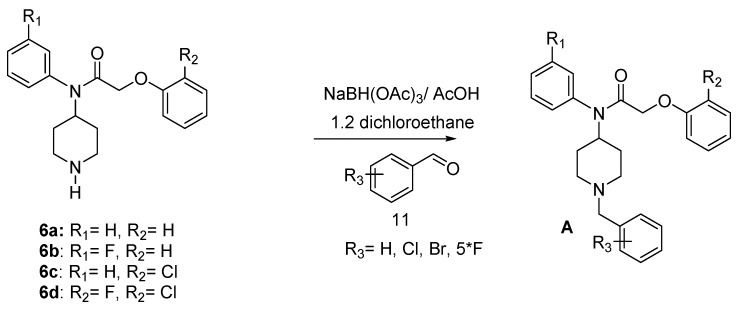
Synthesis of target compounds **A.**

A pharmaco-modulation has been achieved on the parent molecule **6** taking advantage of the nucleophilicity of the piperidine nitrogen leading to compounds **17–34** in good yield. Thus, reductive amination [28,29,30,31] of **6** with benzaldehyde derivatives in 1,2-dichloroethane (ClCH_2_CH_2_Cl), gave compounds **A** (Scheme 4) (Table 2).

### 2.2. The Antimalarial Activity of Derivatives ***6*** and Target Compounds ***A***

Studies have shown that compounds with piperidine rings [4,8,21,22,23,24,25,26] have good selectivity and activity for the *P. falciparum* strain. This prompted us to assess their antiplasmodial activity against the chloroquine-sensitive 3D7 and chloroquine-resistant W2 strains of *P. falciparum* as well as their cytotoxic activity against HUVEC cells (Table 3 and Table 4). Solutions of the 22 synthetic products and the negative control (chloroquine (CQ)) were prepared by two-fold dilution, in a dose-titration range of 0.098–100 µg/mL, to obtain 11 concentrations each, and all of them were inactive against W2 (IC_50_ > 100). The compounds exhibited activities in the nanomolar range against both parasitic strains. Their cytotoxicity against HUVEC ranged from CC_50_ 0.052 ± 0.004 to >100 mM, thus resulting in varied selectivity indexes (SI), 26 for **13b** in the 3D7 strain and >11.3 for **14c** in the W2 strain. Compared with chloroquine (IC_50_ = 22.38 (3D7) and 134.12 (W2)), the compounds **13b** (IC_50_ = 13.30 nM) and **12a** (IC_50_ = 11.06 nM) showed a strong activity against W2. Molecules **13b** (IC_50_ = 4.19 nM), **12d** (IC_50_ = 13.64 nM), **14d** (IC_50_ = 14.85 nM) and **6b** (IC_50_ = 17, 42 nM) had the highest activity against 3D7.

Interestingly, compounds **6c** and **6d** are inactive against both strains. However, after pharmacomodulation on the nitrogen atom, their derivatives **12d**, **14d**, **17c, 13c** and **14c** showed good activity against both strains.

Compound **13b** exhibits 5-fold more activity against strain 3D7 and 10-fold more against strain W2 with very low cytotoxicity (CC_50_ = 112 nM), resulting in a high selectivity index (SI = 26.7 (3D7) and 8.4 (W2), respectively) relative to chloroquine (CC_50_ = 37.56 nM, SI = 1.7 (3D7) and 0.3 (W2)).

Substitution of the piperidine nitrogen atom with a pentafluorobenzyl moiety did not significantly alter the activity of its derivative molecules against both strains. Indeed, the compounds **17b** and **17d,** derived from **6b** and **6d,** respectively, remained inactive while the activity of **17a** (37.63 nM (3D7) and 47.84 nM (W2)) and **17c** (14.65 nM (3D7) and 36.88 nM (W2)) respectively, and **6a** (34.46 nM (3D7) and 61.37 nM (W2)) and **6c** (17.42 nM (3D7) and 30.35 nM (W2)) varied slightly.

## 3. Materials and Methods

### 3.1. Apparatus, Materials, and Analytical Reagents

All chemical reagents and anhydrous solvents were obtained from commercial sources and used without further purification. The ^1^H- and ^13^C-NMR spectra were recorded in CDCl_3_ at ambient temperature on an AMX 500 spectrometer (Bruker, Palaiseau, France). Some product structures were confirmed by DEPT 135, HMQC and HMBC experiments. Chemical shifts are given in δ (ppm) and coupling constants *J* (Hz) relative to TMS used as internal standard; multiplicities were recorded as s (singlet), d (doublet), dd (double doublet), t (triplet), dt (double triple), q (quartet) or m (multiplet). Reactions involving anhydrous conditions were conducted in dry glassware under a nitrogen atmosphere. The infrared spectra have been recorded on a model 842 spectrometer (Perkin-Elmer, 842) using polystyrene as reference. The melting points have been measured on a Tottoli S Bucchi device (Buchi, Rungis, France). Microanalysis have been done on a Perkin-Elmer 2400-CMN apparatus (Perkin ElmerVillebon-sur-Yvette, France). GC/MS conditions: Analyses were performed using a 5890 gas chromatogram connected to a G 1019 A mass spectrometer (both from Hewlett Packard, Alpharetta, GA, USA) operating in the electrospray ionization mode (ESI).

### 3.2. Chemistry

#### 3.2.1. General procedure for the Synthesis of *tert*-Butyl 4-(phenylamino) Piperidine-1-carboxylates **3a**–**b**

A solution of aniline (1 equiv) in 1,2-dichloroethane (100 mL) containing *t*-butyl-4-oxo-1-piperidine carboxylate (1 equiv), sodium triacetoxyborohydride (1.5 equiv) and acetic acid (1.5 equiv) was stirred for 24 h at 20 °C. 1N NaOH (50 mL, 50 mmol) and 50 mL of ethyl acetate were added. The phases were separated and the aqueous layer was extracted with ethyl acetate (3x25 mL). The combined organic layers were dried over MgSO_4,_ filtered and concentrated under reduced pressure. The residue was purified by crystallization (ether petroleum/ethyl acetate (8:2)).

*tert-Butyl 4-(phenylamino) piperidine-1-carboxylate* (**3a**). Aniline (2.8 g, 30.11 mmol) in 1.2-dichloroethane (100 mL) containing *t*-butyl-4-oxo-1-piperidine carboxylate (6 g, 30.11 mmol), sodium triacetoxyborohydride (9.57 g, 45.1 mmol) and acetic acid (2.71 g, 45.16 mmol) gave compound **3a** (7.07 g, 85%), m.p.: 105 °C. ^1^H-NMR (CDCl_3_): 1.3 (m, 2H, CH_2_); 1.49 (s, 9H, 3CH_3_); 2.0 (m, 2H, CH_2_); 2.9 (m, 2H, CH_2_); 3.3 (m, 1H, CH); 3.7 (broadband, 1H, NH); 4.1 (m, 2H, CH2); 6.5–7.5 (m, 5H aromatic). ^13^C-NMR (CDCl_3_) δ: 28.57 (3× CH_3_); 32.52 (2× CH_2_); 42.30 (2× CH_2_); 50.22 (CH); 79.72 (C); 113.42 (2× CHAr); 117.61 (CHAr); 129.49 (2× CHAr); 149.89 (C); 154.92 (C). MS (*m*/*z*): calcd. for C_16_H_24_N_2_O_2_ 276.2 found 277.1 [M + 1]; IR cm^−1^: 1763.07 (CO carbamate); 1671.6 (CO, amide).

*tert-Butyl 4-(3-fluorophenylamino) piperidine-1-carboxylate* (**3b**). 3-Fluoroaniline (3.34 g, 30.11 mmol) in 1,2-dichloroethane (100 mL) containing *t*-butyl-4-oxo-1-piperidine carboxylate (6 g, 30.11 mmol), sodium triacetoxyborohydride (9.57 g, 45.1 mmol) and acetic acid (2.71 g, 45.16 mmol) gave compound **3b** (6.58 g, 74%); m.p.: 114 °C. ^1^H-NMR (CDCl_3_): 1.3 (m, 2H, CH_2_); 1.49 (s, 9H, 3CH_3_); 2.0 (m, 2H, CH_2_); 2.9 (m, 2H, CH_2_); 3,4 (m, 1H, CH); 3.8 broad band, 1H, NH); 4.1 (m, 2H, CH_2_); 6.5–7.5 (m, 4H aromatic). ^13^C-NMR (CDCl_3_) δ: 28.56 (3× CH_3_); 32.36 (2× CH_2_); 42.73 (2× CH_2_); 50.26 (CH); 79.81 (C); 99.95 (d, *J* = 25.26 Hz, CHAr); 103.84 (d, *J* = 21.25 Hz, CHAr); 109.19 (d, *J* = 2.2 Hz, CHAr); 130.58 (d, *J* = 10.30 Hz, CHAr); 148.73 (d, *J* = 10.55 Hz, C); 154.896 (C), 163.34 (d, *J* = 242.72 Hz, C). ESI (*m*/*z*) calcd for C_16_H_23_FN_2_O_2_ 294.2; found 294.1 [M + 1]; IR cm^−1^: 1760.07 (CO carbamate); 1670.6 (CO, amide).

#### 3.2.2. General Procedure for the Coupling with Phenoxyacetyl chloride: Synthesis of Compounds **5a**–**b**

To an ice-cooled suspension of sodium hydride (60% in mineral oil, 2 equiv) in CH_2_Cl_2_ (10 mL) was added dropwise a solution of compound **3** (1 equiv), in CH_2_Cl_2_ (15 mL). After stirring 15 min phenoxyacetyl chloride **4** (2 equiv) was added. The reaction mixture was stirred for 1 h at 0 °C, and the temperature was raised to room temperature during 3 h. 20 mL of saturated solution of NaHCO_3_ was carefully added. The aqueous layer was extracted with CH_2_Cl_2_ (3 × 20 mL). The combined organic phases were dried over MgSO_4_, and concentrated in vacuum. The crude product was purified by crystallisation (ether petroleum/ethyl acetate (8:2)).

*tert-Butyl 4-(2-phenoxy-N-phenylacetamido) piperidine-1-carboxylate* (**5a**). Following the general procedure, sodium hydride (60% in mineral oil, 0.723 g, 18.1 mmol) in CH_2_Cl_2_ (10 mL) was added dropwise a solution of compound **3a** (2.5 g, 9.05 mmol), in CH_2_Cl_2_ (15 mL). After stirring 15 min phenoxyacetyl chloride (2.5 mL, 18.1 mmol) was added to give compound **5a** (3.06 g, 82%). ^1^H-NMR (CDCl_3_): 1.25 (m, 2H, CH_2_); 1.4 (s, 9H); 1.8 (m, 2H, CH_2_); 2.9 (m, 2H, CH_2_); 4.1 (m, 2H, CH_2_); 4.25 (m, 2H, CH_2_); 4.8 (m, 1H, CH); 6.7–7.5 (m, 10H aromatic). ^13^C-NMR (CDCl_3_) δ: 28.456 (3× CH_3_, C(CH_3_)_3_); 30.32 (2× CH_2_); 43.315 (2× CH_2_); 52.96 (CH); 66.7 (CH2); 79.73 (C); 114.8 (2× CHAr); 121.47 (CHAr); 129.34 (CH); 129.46 (2× CHAr); 129.88 (2× CHAr); 130.09 (2× CHAr); 136.89 (C); 154.63 (C); 158.14 (C); 167.6 (C). ESI (*m*/*z*) calcd for C24H30N2O4 410, 2 found 411.1 [M + 1], IR cm^−1^: 1744.95 (CO carbamate); 1652.06 (CO, amide).

*tert-Butyl 4-(N-(3-fluorophenyl)-2-phenoxyacetamido) piperidine-1-carboxylate* (**5b**). Following the general procedure, sodium hydride (60% in mineral oil, 0.677 g, 16.93 mmol) in CH_2_Cl_2_ (10 mL) was added dropwise a solution of compound **3b** (2.49 g, 8.46 mmol), in CH_2_Cl_2_ (15 mL). After stirring 15 min phenoxyacetyl chloride (2.88 g, 16.93 mmol) was added to give compound **5b** (2.34 g, 65%). ^1^H-NMR (CDCl_3_): 1.25 (m, 2H, CH_2_); 1.4 (s, 9H, 3× CH_3_); 1.8 (d, 2H, CH_2_); 2.8 (m, 2H, CH_2_); 4.15 (m, 2H, CH_2_); 4.30 (s, 2H, CH_2_); 4.75 (m, H, CH); 6.7–7.6 (m, 9 H aromatic). ^13^C-NMR (CDCl_3_) δ: 28.44 (3× CH_3_, C(CH_3_)_3_); 30.284 (2× CH_2_); 43.16 (2× CH_2_); 53.188 (CH); 66.82 (CH_2_); 79.81 (C); 114.76 (2× CHAr); 116.53 (d, *J* = 20.74 Hz, CHAr); 117.63 (d, *J* = 21.49 Hz, CHAr); 121.60 (CHAr); 125.97 (d, *J* = 3.14 Hz, CHAr); 129.50 (2× CHAr);130.92 (d, *J* = 7.3 Hz, CHAr); 138.52 (d, *J* = 9.17 Hz, C); 154.58 (C); 157.97 (C); 161.91 (d, *J* = 250.14 Hz, C); 167.19 (C). ESI (*m*/*z*): calcd for C_24_H_29_FN_2_O_4_ 428.2, found 429.0 [M + 1]; IR cm^−1^: 1745.07 (CO carbamate); 1660.6 (CO, amide).

#### 3.2.3. General Procedure for the Coupling with Chloroacetyl chloride: Synthesis of Compounds **7a**–**b**

One equiv of compound **3** was dissolved in 25 mL of CH_2_Cl_2_, 2 equiv. of potassium carbonate were added and the mixture was cooled to 0 °C. Two equiv. of chloroacetyl chloride were added to 0 °C, and the mixture was stirred overnight. The reaction was quenched by addition of a saturated solution of NaHCO_3_ (25 mL), the aqueous phase was decanted and extracted twice with 15 mL of CH_2_Cl_2_. Combined organic phases were washed with water and brine, dried over MgSO_4_ and concentrated in vacuum. The crude product was purified by crystallisation (ether petroleum/ethyl acetate (8:2)).

*tert-Butyl-4-(2-chloro-N-phenylacetamido) piperidine-1-carboxylate* (**7a**). Following the general procedure, 2.5 g (9.03 mmol) of compound **3a** were dissolved in 25 mL of CH_2_Cl_2_, 2.5 g (18.06 mmol) of potassium carbonate were added and the mixture was cooled to 0 °C. 2.04 g (18.06 mmol) of chloroacetyl chloride were added, and the mixture was stirred overnight to afford **7a** as a white solid (2.55 g, 80%), m.p.: 110 °C. 1H-NMR (CDCl3, 500 MHz): 1.25 (m, 2H, CH2); 1.4 (s, 9 H, 3× CH3); 1.8 (m, 2H, CH2); 3.7 (s, 2H, CH2); 2.8 (m, 2H, CH2); 4.1 (m, 2H, CH2); 4.75 (m, 1H, CH); 7–7.5 (m, 5H aromatic). ^13^C-NMR (125 MHz, CDCl_3_) δ: 28.485 (C(CH_3_)_3_); 30.32 (2× CH_2_); 42.55 (CH_2_); 43.41 (2× CH_2_); 53.5 (CH); 79.8 (C); 129.47 (2× CHAr); 129.9 (CHAr); 130.2 (2× CH); 137.3 (C); 154.64 (C); 166.04 (C). ESI (*m*/*z*): calcd for C_18_H_25_ClN_2_O_3_ 352.2, found 353.0 [M + 1]; IR cm^−1^: 1730.5 (CO carbamate); 1699.03 (CO, amide).

*tert-Butyl 4-(2-chloro-N-(3-fluorophenyl) acetamido)piperidine-1-carboxylate* (**7b**). Following the general procedure, 2.5 g (8.46 mmol) of compound **3b** were dissolved in 25 mL of CH_2_Cl_2_, then 2.34 g (16.93 mmol) of potassium carbonate were added and the mixture was cooled to 0 °C. Chloroacetyl chloride (1.91 g, 18.06 mmol) was added, and the mixture was stirred overnight to afford **7b** (2.66 g, 85%), m.p.: 90 °C. ^1^H-NMR (CDCl_3_, 500 MHz): 1.25 (m, 2H, CH_2_); 1.4 (s, 9 H, 3CH_3_); 1.8 (m, 2H, CH_2_); 3.7 (s, 2H, CH_2_); 2.8 (m, 2H, CH_2_); 4.1 (m, 2H, CH_2_); 4.75 (m, 1H, CH); 7–7.5 (m, 5H aromatic). ^13^C-NMR (125 MHz, CDCl_3_) δ: 28.44 (3× CH_3_); 30.27 (2× CH_2_); 42.26 (CH_2_); 42.97 (2× CH_2_); 53.69 (CH); 79.878 (C); 116.72 (d, *J* = 20.74 Hz, CHAr); 117.79 (d, *J* = 21.49 Hz, CHAr); 126.14 (CHAr); 131.08 (d, *J* = 9.17 Hz, CHAr); 138.8 (d, *J* = 9.17 Hz, C); 154.58 (C); 161.926 (C); 163.92 (d, *J* = 234,80 Hz, C). ESI (*m*/*z*): calcd for C_18_H_24_ClFN_2_O_3_ 370.1 found 371.0 [M + 1]; IR cm^−1^: 1730.5 (CO carbamate); 1699.08 (CO, amide).

#### 3.2.4. General Procedure for Synthesis of Compounds **9a**–**b**

To a mixed solution of acetonitrile/acetone (50/50) were added 1 equiv of compound **7**; 1 equiv of 2-chlorophenol and 2 equiv of potassium carbonate. After 12 h of stirring under reflux the mixture was concentrated in vacuum. The residual was dissolved in 15 mL of ethyl acetate and (1N) of NaOH (15 mL), the aqueous phase was decanted and extracted twice with 15 mL of ethyl acetate. Combined organic phases were washed with water and brine, dried over MgSO_4_ and concentrated in vacuum. The crude product was purified by crystallisation (ether petroleum/ethyl acetate (8:2)).

*tert-Butyl 4-(2-(2-chlorophenoxy)-N-phenylacetamido) piperidine-1-carboxylate* (**9a**). Following the general procedure, 2.83 g (8.04 mmol) of compound **7**; 1.032 g (8.04 mmol) of 2-chlorophenol and 2.22 g (16.09 mmol) of potassium carbonate. (3.57 g, 99%); m.p.: 123 °C; ^1^H-NMR (CDCl_3_, 600 MHz): 1.3 (m, 2H, CH2); 1.5 (s, 9H, 3× CH_3_); 1.80 (m, 2H, CH_2_); 2.85 (m, 2H, CH_2_); 4.12 (m, 1H, CH); 4.33 (s, 2H, CH2); 4.76 (m, H, CH); 6.4–7.5 (m, 9H aromatic). ^13^C-NMR (150 MHz, CDCl_3_) δ: 28.46(3× CH_3_); 29.83 (2× CH_2_); 42.75 (2× CH_2_) 53.04 (CH); 67.62 (CH_2_); 79.78 (C); 113.95 (2× CHAr); 122.27 (2× CHAr); 123.27 (C); 127.61 (CHAr); 129.45(CHAr); 129.97 (CHAr); 130.14 (CHAr); 130.45 (CHAr); 136.64 (C); 153.9 (C); 154.64 (C); 166.84 (C). ESI (*m*/*z*): calcd for C_24_H_29_ClN_2_O_4_ 444.1, found 445.0 [M + 1]; IR cm^−1^: 1727.5 (CO carbamate); 1693.33 (CO, amide).

*tert-Butyl 4-(2-(2-chlorophenoxy)-N-(3-fluorophenyl) acetamido) piperidine-1-carboxylate* (**9b**). Following the general procedure, 1.80 g (4.87 mmol) of compound **7**; 0.626 g (4.87 mmol) of 2-chlorophenol and 1.34 g (9.75 mmol) of potassium carbonate were reacted to give **9b** (2.01 g, 89%); m.p.: 125°C; ^1^H-NMR (CDCl_3_, 500 MHz): 1.18 (m, 2H, CH_2_); 1.21 (s, 9H, 3CH_3_); 1.74 (m, 2H, CH_2_); 2.71 (t, *J* = 12 Hz, 2H, CH_2_); 4.06 (m, 2H, CH_2_); 4.31 (s, 2H, CH_2_); 4.67 (m, 1H, CH) 6.4–7.5 (m, 8H aromatic). ^13^C-NMR (125 MHz, CDCl_3_) δ: 27.12 (2× CH_2_); 28.36 (3CH_3_); 30.21 (2× CH_2_); 53.32 (CH); 67.75 (CH_2_); 79.75 (C); 114.0 (CHAr); 116.43 (d, *J* = 31.25 Hz, CHAr); 117.40 (d, *J* = 26.25 Hz, CHAr); 122.36 (CHAr); 123.27 (C); 125.94 (CHAr); 127.55 (CHAr); 130.52 (CHAr); 130.84 (d, *J* = 11.25 Hz, CHAr); 138.20 (d, *J* = 20 Hz, C); 153.69 (C); 154.51 (C); 161.41 (d, *J* = 281.25 Hz, C); 166.60 (C). ESI (*m*/*z*): calcd for C_24_H_28_ClFN_2_O_4_ 462.2, found 463.0 [M + 1]; IR cm^−1^: 1727.5 (CO carbamate); 1693.33 (CO, amide).

#### 3.2.5. General Procedure for Deprotection: Synthesis of **6a**–**b**

One equiv of compound **5** was dissolved in 15 mL of CH_2_Cl_2_, and 13 equiv of trifluoroacetic acid were added. After 2 h of stirring at room temperature, the reaction mixture was concentrated under vacuum. The residue was dissolved in 5 mL of ethyl acetate then neutralized with NaHCO_3_ (5%). The aqueous layer was extracted with ethyl acetate (4x5mL). The combined organic phases were dried over MgSO_4_, filtered and concentrated under reduced pressure. The crude product was purified by crystallisation (ether petroleum/ethyl acetate (8:2)).

*2-Phenoxy-N-Phenyl-N-(piperidin-4-yl) acetamide* (**6a**). Following the general procedure, 3.06 g (7.48 mmol) of compound **5a** were dissolved in 15 mL of CH_2_Cl_2_, then 7.49 mL (97.24 mmol) of trifluoroacetic acid were added to produce compound **6a** (1.15 g, 50%); m.p.: 59 °C. ^1^H-NMR (CDCl_3_, 600 MHz): 1.5 (m, 2H, CH_2_); 1.9 (m, 2H, CH_2_); 2.7 (m, 2H, CH_2_); 3.4 (m, 2H, CH_2_); 3.4 (broad, 1H, NH); 4.6 (s, 2H, O-CH_2_); 4.75 (m, 1H, CH), 6.5–7.7 (m, 10H aromatic). ^13^C-NMR (150 MHz, CDCl_3_) δ: 30 (2× CH_2_); 45.06 (2× CH_2_); 52.15 (CH); 66.74 (CH_2_); 114.76 (2× CHAr); 121.53 (CHAr); 129.17 (CHAr); 129.46 (2× CHAr); 129.50 (CHAr); 130.00 (2× CHAr); 130.17 (CHAr); 136.63 (C); 158.07 (C); 167.48 (C). ESI (*m*/*z*): calcd for C_19_H_22_N_2_O_2_ 310.2, found 311.0 [M + 1]; IR cm^−1^: 3443 (NH), 1691.33 (CO).

*N-(3-Fluorophenyl)-2-phenoxy-N-(piperidin-4-yl) acetamide* (**6b**). Following the general procedure, 4.65 g (10.87 mmol) of compound **5b** were dissolved in 15 mL of CH_2_Cl_2_, 10.81 mL (141.31 mmol) of trifluoroacetique acid were added. (2.5 g, 70%); m.p.: 200 °C; ^1^H-NMR (CDCl_3_, 500 MHz): 1.3 (m, 2H, CH_2_); 1.80 (m, 2H, CH_2_); 2.85 (m, 2H, CH_2_); 3.7 (broad, 1H, NH); 4.06 (m, 2H, CH_2_); 4.31 (m, 2H, CH_2_); 4.67 (m, 1H, CH) 6.4–7.5 (m, 9H aromatic). ^13^C-NMR (125 MHz, CDCl_3_) δ: 27.14 (2× CH_2_); 43.99 (2× CH_2_); 51.11 (CH); 66.69 (CH_2_); 114.74 (2× CHAr); 117.34 (d, *J* = 50.53 Hz, CHAr); 121.97 (CHAr); 129.68 (2× CHAr); 129.86 (CHAr); 131.71 (CHAr); 138.23 (C); 153.69 (C); 164.11–161.27 (C); 168.211 (C). ESI (*m*/*z*): calcd for C_19_H_21_FN_2_O_2_ 328.2; found 329.166 [M + 1]; IR cm^−1^: 3445 (NH), 1693.01 (CO).

*2-(2-Chlorophenoxy)-N-Phenyl-N-(piperidin-4-yl) acetamide* (**6c**). Compound **9** (3.90 g, 8.78 mmol) was dissolved in 15 mL of CH_2_Cl_2_, then 8.73 mL (114.14 mmol) of trifluoroacetic acid were added, to give **6c** (3.02 g, 95%); m.p.: 161 °C; ^1^H-NMR (CDCl_3_, 600 MHz): 1.70 (m, 2H, CH_2_); 1.93 (broad, 1H, NH); 2.06 (m, 2H, CH_2_); 2.97 (m, 2H, CH_2_); 3.38 (m, 2H, CH_2_); 4.37 (s, 2H, CH_2_); 4.77 (m, 1H, CH); 6.5–7.5 (m, 9H Ar). ^13^C-NMR (150 MHz, CDCl_3_) δ: 27.12 (2× CH_2_); 43.55 (2× CH_2_); 50.68 (CH); 67.53 (CH_2_); 114.01 (2× CHAr); 122.27 (CHAr); 123.27 (C); 127.55 (CHAr); 129.55(2× CHAr); 129.84 (CHAr); 130.24 (CHAr); 130.55 (CHAr); 135.76 (C); 153.67 (C); 167.27 (C). ESI (*m*/*z*): calcd for C_19_H_21_ClN_2_O_2_ 344.1; found 345.13 [M + 1]; IR cm^−1^: 3445 (NH), 1691.33 (CO).

*2-(2-Chlorophenoxy)-N-(3-fluorophenyl)-N-(piperidin-4-yl) acetamide* (**6d**). Following the general procedure, 1.99 g (4.327 mmol) of compound **9** were dissolved in 15 mL of CH_2_Cl_2_, and 4.30 mL (56.25 mmol) of trifluoroacetic acid were added to afford **6d** (2.4 g 60%); m.p.: 140°C; ^1^H-NMR (CDCl_3_, 600 MHz): 1.40 (m, 2H, CH_2_); 2.06 (m, 2H, CH_2_); 2.7 (broad band, 1H, NH) 2.97 (m, 2H, CH_2_); 3.2 (m, 2H, CH_2_); 4.37 (s, 2H, CH_2_); 4.77 (m, 1H, CH); 6.5–7.5 (m, 8H aromatic). ^13^C-NMR (150 MHz, CDCl_3_) δ: 30.9 (2× CH_2_); 45.7 (2× CH_2_); 53.11 (CH); 67.79 (CH_2_); 114.05 (CHAr); 116.55 (d, *J* = 24.9 Hz, CHAr); 117.74 (d, *J* = 106.5 Hz, CHAr); 122.447 (CHAr); 123.27 (CHAr); 126.068 (d, *J* = 3.75 Hz, CHAr); 127.673 (CHAr); 130.619 (CHAr); 131.958 (d, *J* = 10.95 Hz, CHAr); 138.292 (C); 153.780 (C); 161.963 (d, *J* = 298.5 Hz, C); 166.60 (C). ESI (*m*/*z*): calcd for C_19_H_20_ClFN_2_O_2_ 362.1; found 363.12 [M + 1]; IR cm^−1^: 3445 (NH), 1691.33 (CO).

#### 3.2.6. General Procedure for Synthesis of Target Compounds **A**

A solution of benzaldehyde derivatives (1 equiv) in 1,2-dichloroethane containing compound **6** (1 equiv), sodium triacetoxyborohydride (1.5 equiv) and acetic acid (1.5 equiv) was stirred for 24 h at 20 °C. 1N NaOH (15 mL) and 15 mL of ethyl acetate were added. The phases were separated and the aqueous layer was extracted with ethyl acetate (3 × 15 mL). The combined organic layers were dried over MgSO_4,_ filtered and concentrated under reduced pressure. The residue was purified by chromatography on silica gel (petroleum ether/EtOAc (8:2)).

*N-(1-Benzylpiperidin-4-yl)-2-phenoxy-N-phenylacetamide* (**12a**). Following the general procedure for reductive amination, using benzaldehyde derivative **11** (R_3_ = H) (34 mg, 0.322 mmol); compound **6a** (100 mg, 0.322 mmol); sodium triacetoxyborohydride (102 mg, 0.4838 mmol) and acetic acid (29 mg, 0.4838 mmol) in 1,2-dichloroethane (5 mL) give compound **12a** (0.1291 g, 52%); m.p. 89 °C. ^1^H-NMR (CDCl_3_, 600 MHz): 1.32 (m, 2H, CH_2_); 1.74 (m, 2H, CH_2_); 2.06 (m, 2H, CH_2_); 2.83 (m, 2H, CH_2_); 3.42 (s, 2H, CH_2_); 4.15 (s, 2H, CH_2_); 4.62 (m, H, CH); 6.68–7.38 (m, 15H aromatic); ^13^C-NMR (150 MHz, CDCl_3_) δ: 32.17 (2× CH2); 43.72 (2× CH_2_); 52.84 (CH); 62.89 (CH_2_); 66.73 (CH_2_); 114.74 (4× CHAr); 121.32 (CHAr); 128.25 (CHAr); 129.08 (CHAr); 129.34 (4× CHAr); 129.68 (2× CHAr); 130.14 (2× CHAr); 135.30 (C) 136.69 (C); 158.14 (C); 167.25 (C). ESI (*m*/*z*): calcd for C_26_H_28_N_2_O_2_ 400.02; found 401.22 [M + 1]; IR cm^−1^: 1680 (CO).

*N-(1-Benzylpiperidin-4-yl)-N-(3-fluorophenyl)-2-phenoxyacetamide* (**12b**). Following the general procedure, benzaldehyde derivative **11** (R_3_ = H(*o*)) (48.4 mg, 0.457 mmol); compound **6b** (150 mg, 0.457 mmol); sodium triacetoxyborohydride (145.3 mg, 0.6855 mmol) and acetic acid (41.6 mg, 0.6855 mmol) in 1.2-dichloroethane (5 mL) was stirred for 24 h at 20 °C to furnish **12b** (0.103 g, 54%); m.p.: 90 °C. ^1^H-NMR (CDCl_3_, 500 MHz): 1.36 (m, 2H, CH_2_); 1.83 (m, 2H, CH_2_); 2.15 (m, 2H, CH_2_); 2.94 (m, 2H, CH_2_); 3.50 (s, 2H, CH_2,_); 4.30 (s, 2H, CH_2_); 4.67 (m, H, CH); 6.78–7.47 (m, 14H aromatic). ^13^C-NMR (125 MHz, CDCl_3_) δ: 30.26 (2× CH2); 43.72 (2× CH_2_); 52.84 (CH); 62.80 (CH_2_); 66.81 (CH_2_); 114.73 (4× CHAr); 117.50 (d, *J* = 22 Hz, CHAr); 121.47 (2× CHAr); 126.03 (CHAr); 127.16 (C); 128.24 (2× CHAr); 129.16 (CHAr); 129.40 (2× CHAr); 130.66 (CHAr); 138.67 (C); 157.99 (C); 161.59 (d, *J* = 251 Hz, C); 167.06 (C). ESI (*m*/*z*): calcd for C_26_H_27_FN_2_O_2_ 418.2; found 419.21 [M + 1]; IR cm^−1^: 1687.

*2-(2-Chlorophenoxy)-N-(1-benzylpiperidin-4-yl)-N-phenylacetamide* (**12c**). Following the general procedure, benzaldehyde derivative **11** (R_3_ = H) (46.2 mg, 0.435 mmol); compound **6c** (150 mg, 0.435 mmol); sodium triacetoxyborohydride (138.5 mg, 0.6538 mmol) and acetic acid (39.5 mg, 0.6538 mmol) in 1.2-dichloroethane (5 mL) was stirred for 24 h at 20 °C to afford **12c** (0.0946 g, 50%); m.p.: 83 °C; ^1^H-NMR (CDCl_3_, 400 MHz): 1.47 (m, 2H, CH_2_); 1.84 (m, 2H, CH_2_); 2.14 (m, 2H, CH_2_); 2.94 (m, 2H, CH_2_); 3.48 (s, 2H, CH_2_); 4.35 (s, 2H, CH_2_); 4.64 (m, H, CH); 6.73–7.47 (m, 14H aromatic). ^13^C-NMR (100 MHz, CDCl_3_) δ: 30.23 (2× CH2); 43.37 (2× CH_2_); 52.88 (CH); 62.90 (CH_2_); 67.66 (CH_2_); 114.01 (2× CHAr); 122.09 (2× CHAr).; 123.26 (C); 127.11 (C); 127.46 (2× CHAr); 128,21 (2× CHAr); 129.12 (CHAr); 129.71 (2× CHAr); 130.18 (2× CHAr); 130.41 (CHAr); 136.99 (C); 153.94 (C); 166.67 (C). ESI (*m*/*z*): calcd for C_26_H_27_ClN_2_O_2_ 434.2; found 435.18 [M + 1]; IR cm^−1^: 1683 (CO).

*2-(2-Chlorophenoxy)-N-(1-benzylpiperidin-4-yl)-N-(3-fluorophenyl) acetamide* (**12d**). Following the general procedure, benzaldehyde derivatives **11** (R_3_ = H(*o*)) (43.91 mg, 0.4142 mmol); compound **6d** (150, 0.4142 mmol); sodium triacetoxyborohydride (131.6 mg, 0.6213 mmol) and acetic acid (37.3 mg, 0.6213 mmol) in 1,2-dichloroethane (5 mL) was stirred for 24 h at 20 °C, giving **12d** (0.0936 g, 54%); m.p.: 73 °C. ^1^H-NMR (CDCl_3_, 600 MHz): 1.25 (m, 2H, CH_2_); 1.43 (m, 2H, CH_2_); 1.78 (m, 2H, CH_2_); 2.14 (m, 2H, CH_2_); 2.92 (s, 2H, CH_2_); 3.49 (s, 2H, CH_2_).; 4.36 (s, 2H, CH_2_); 4.63 (m, H, CH); 6.73–7.47 (m, 13H aromatic). ^13^C-NMR (150 MHz, CDCl_3_) δ: 30.12 (2× CH_2_); 43.37 (2× CH_2_); 52.82 (CH); 62.90 (CH_2_); 67.77 (CH_2_); 114.03 (2× CHAr); 116.50 (CHAr); 116.67 (C); 117.63 (d, *J* = 21.36 Hz, CHAr); 122.39 (2× CHAr); 123.31 (C); 126.11 (d, *J* = 2.51 Hz, CHAr); 127.65 (CHAr); 128.43 (CHAr); 129.41 (CHAr); 130.60 (2× CHAr); 130.93 (d, *J* = 8.79 Hz, CHAr); 138.36 (C); 153.81 (C); 161.95 (d, *J* = 250.14 Hz, C); 166.67 (C). ESI (*m*/*z*): calcd for C_26_H_26_ClFN_2_O_2_ 452.2; found 453.18 [M + 1]; IR cm^−1^: 1689 (CO).

*N-(1-(2-Bromobenzyl)piperidin-4-yl)-2-phenoxy-N-phenylacetamide* (**13a**). Following the general procedure for reductive amination using benzaldehyde derivative **11** (R_3_ = Br (*o*)) (75.3 mg, 0.410 mmol); compound **6a** (127 mg, 0.410 mmol); sodium triacetoxyborohydride (130.15 mg, 0.615 mmol) and acetic acid (36.9 mg, 0.615 mmol)) in 1,2-dichloroethane (5 mL) to give compound **13a** (0.119 g, 60%); m.p. 66°C. ^1^H-NMR (CDCl_3_, 600 MHz): 1.44 (m, 2H, CH_2_); 1.64 (m, 2H, CH_2_); 2.25 (m, 2H, CH_2_); 2.90 (m, 2H, CH_2_); 3.53 (s, 2H, CH_2,_); 4.23 (s, 2H, CH_2_); 4.70 (m, H, CH); 6.73–7.47 (m, 14H aromatic). ^13^C-NMR (150 MHz, CDCl_3_) δ: 30.50 (2× CH_2_); 53.07 (CH); 53.13 (2× CH_2_); 61.77 (CH_2_); 66.77 (CH_2_); 114.80 (2× CHAr); 121.42 (CHAr); 124.69 (C); 127.27 (CHAr); 128.44 (CHAr); 129.20 (CHAr); 129.46 (2× CHAr); 129.82 (2× CHAr); 130.24 (2× CHAr); 130.64 (CHAr); 132.82 (CHAr); 137.12 (C); 137.21 (C); 158.19 (C); 167.33 (C). ESI (*m*/*z*): calcd for C_26_H_27_BrN_2_O_2_ 478.1; found 479.13 [M + 1]; IR cm^−1^: 1678 (CO).

*N-(1-(2-Bromobenzyl)piperidin-4-yl)-N-(3-fluorophenyl)-2-phenoxyacetamide* (**13b**). Following the general procedure, benzaldehyde derivatives **11** (R_3_ = Br (*o*)) (84.5 mg; 0.457 mmol); compound **6b** (150 mg, 0.457 mmol); sodium triacetoxyborohydride (142.29 mg, 0.685 mmol) and acetic acid (41.16 mg, 0.685 mmol) in 1,2-dichloroethane (5 mL) was stirred for 24 h at 20 °C. Yield of **13b**: 0.176 g (58%); IR cm^−1^: 1687; MS: 469.14 [M + 1]; m.p.: 55 °C; H^1^-NMR (CDCl_3_, 500 MHz): 1.36 (m, 2H, CH_2_); 1.83 (m, 2H, CH_2_); 2.15 (m, 2H, CH_2_); 2.94 (m, 2H, CH_2_); 3.50 (s, 2H, CH_2,_); 4.30 (s, 2H, CH_2_); 4.67 (m, H, CH); 6.78–7.47 (m, 13H aromatic). ^13^C-NMR (125 MHz, CDCl_3_) δ: 30.26 (2× CH_2_); 42.89 (2× CH_2_); 52.98 (CH); 61.65 (CH_2_); 66.88 (CH_2_); 114.81 (4× CHAr); 116.42 (d, *J* = 20 Hz, CHAr); 117.52 (d, *J* = 20 Hz, CHAr); 121.47 (2× CHAr); 126.06 (CHAr); 128.56 (C); 129.53 (4× CHAr); 130.22 (d, *J* = 11,25 Hz, C); 132.90 (CHAr); 138.73 (C); 158.05 (C); 161.59 (d, *J* = 247,5 Hz, C); 167.23 (C). ESI (*m*/*z*): calcd for C_26_H_26_BrFN_2_O_2_ 496.1; found 497.14 [M + 1]. IR cm^−1^: 1687 (CO).

*N-(1-(2-Bromobenzyl)piperidin-4-yl)-2-(2-chlorophenoxy)-N-phenylacetamide* (**13c**). Following the general procedure, benzaldehyde derivatives **11** (R_3_ = Br (*o*)) (80.5 mg; 0.435 mmol); compound **6c** (150 mg, 0.435 mmol); sodium triacetoxyborohydride (138 mg, 0.653 mmol) and acetic acid (39.2 mg, 0.653 mmol) in 1,2-dichloroethane (5 mL) was stirred for 24 h at 20 °C to give **13c** (0.122 g, 55%); m.p.: 64 °C. ^1^H-NMR (CDCl_3_, 400 MHz): 1.36 (m, 2H, CH_2_); 1.86 (m, 2H, CH_2_); 2.27 (m, 2H, CH_2_); 2.94 (m, 2H, CH_2_); 3.57 (s, 2H, CH_2_); 4.35 (s, 2H, CH_2_); 4.742 (m, H, CH); 6.73–747 (m, 13H aromatic). ^13^C-NMR (100 MHz, CDCl_3_) δ: 30.34 (2× CH_2_); 42.98 (2× CH_2_); 52.97 (CH); 61.63 (CH_2_); 67.66 (2H, CH_2_); 113.99 (2× CHAr); 122.10 (CHAr); 123.35 (C); 124.62 (C); 127.21 (C); 127.47 (2× CHAr); 128.82 (2× CHAr); 129.74 (2× CHAr); 130.14 (CHAr); 130.42 (CHAr); 132.72 (2× CHAr); 136.91 (C); 153.93 (C); 166.70 (C). ESI (*m*/*z*): calcd for C_26_H_6_BrClN_2_O_2_ 512.1; found 513.09 [M + 1]; IR cm^−1^: 1651 (CO).

*N-(1-(2-Bromobenzyl)piperidin-yl)-2-(2-chlorophenoxy)-N-(3-fluorophenyl) acetamide* (**13d**). Following the general procedure, benzaldehyde derivative **11** (R_3_ = Br (*o*)) (76.63 mg, 0.4142 mmol); compound **6d** (150 mg, 0.4142 mmol); sodium triacetoxyborohydride (131.6 mg, 0.6213 mmol) and acetic acid (37.3 mg, 0.6213 mmol) in 1,2-dichloroethane (5 mL) was stirred for 24 h at 20 °C to furnish **13d** (0.1295 g, 59%); m.p.: 100 °C; ^1^H-NMR (CDCl_3_, 600 MHz): 1.33 (m, 2H, CH_2_); 1.80 (m, 2H, CH_2_); 2.23 (m, 2H, CH_2_); 2.92 (m, 2H, CH_2_); 3.54 (s, 2H, CH_2,_); 4.37 (s, 2H, CH_2_); 4.74 (m, H, CH); 6.73–7.47 (m, 12H aromatic). ^13^C-NMR (150 MHz, CDCl_3_) δ: 30.45 (2× CH_2_); 43.41 (2× CH_2_); 53.04 (CH); 61.72 (CH_2_); 67.74 (CH_2_); 113.95 (2× CHAr); 116.50 (d, *J* = 21 Hz, CHAr); 117.78 (d, *J* = 21 Hz, CHAr); 122.38 (CHAr); 123.25 (C); 124.70 (C); 126.17 (CHAr); 127.32 (CHAr); 127.66 (CHAr); 128.51 (CHAr); 130.60 (CHAr); 130.92 (d, *J* = 9 Hz, CHAr); 132.86 (CHAr);138.60 (C); 138.51 (C); 153.78 (C); 162.10 (d, *J* = 249 Hz, C); 166.68 (C). ESI (*m*/*z*): calcd for C_26_H_25_BrClFN_2_O_2_ 530.1; found 531.08 [M + 1]; IR cm^−1^: 1689 (CO).

*N-(1-(2-Chlorobenzyl)piperidin-4-yl)-2-phenoxy-N-phenylacetamide* (**14a**). Following the general procedure for reductive amination using benzaldehyde derivative **11** (R_3_ = Cl (*o*)) (43.5 mg, 0.4838 mmol); compound **6a** (150 mg, 0.4838 mmol); sodium triacetoxyborohydride (153.8 mg, 0.7253 mmol) and acetic acid (43.5 mg, 0.7253 mmol) in 1,2-dichloroethane (5 mL) gave title compound **14a** (0.109 g, 52%); m.p.: 69 °C. ^1^H-NMR (CDCl_3_, 500 MHz): 1.47 (m, 2H, CH_2_); 1.81 (m, 2H, CH_2_); 2.28 (m, 2H, CH_2_); 2.94 (m, 2H, CH_2_); 3.59 (s, 2H, CH_2_); 4.23 (s, 2H, CH_2_); 4.67 (m, H, CH); 6.73–7.47 (m, 14H aromatic). ^13^C-NMR (125 MHz, CDCl_3_) δ: 30.32 (2× CH_2_); 42.30 (2× CH_2_); 53.03 (CH_2_); 66.82 (CH_2_); 114.84 (4× CHAr); 121.45 (2× CHAr); 126.73 (C); 129.25 (CHAr); 129.47 (4× CHAr); 129.58 (CHAr): 129.85 (CHAr); 130.20 (CHAr); 134.50 (C); 137.02 (C); 158.22 (C); 167.40 (C). ESI (*m*/*z*): calcd for C_26_H_27_ClN_2_O_2_ 434.2; found 435.18 [M + 1]; IR cm^−1^: 1673 (CO).

*N-(1-(2-Chlorobenzylpiperidin-4-yl)-2-(2-chlorophenoxy)-N-phenylacetamide* (**14c**). Following the general procedure, benzaldehyde derivative **11** (R_3_ = Cl (*o*)) (61.02 mg; 0.4359 mmol); compound **6c** (150 mg, 0.4359 mmol); sodium triacetoxyborohydride (138.58 mg, 0.6538 mmol) and acetic acid (39.26 mg, 0.6538 mmol) in 1,2-dichloroethane (5 mL) was stirred for 24 h at 20 °C to produce **14c** (0.1326 g, 65%); m.p.: 55 °C; ^1^H-NMR (CDCl_3_, 400 MHz): 1.36 (m, 2H, CH_2_); 1.75 (m, 2H, CH_2_); 2.16 (m, 2H, CH_2_); 2.84 (m, 2H, CH_2_); 3.49 (s, 2H, CH_2_); 4.25 (s, 2H, CH_2_); 4.62 (m, H, CH); 6.64–7.37 (m, 13H aromatic). ^13^C-NMR (100 MHz, CDCl_3_) δ: 30.38 (2× CH_2_); 52.99 (CH); 53.03 (2× CH_2_); 59.09 (CH_2_); 67.64 (CH_2_); 113.97 (2× CHAr); 122.10 (2× CHAr); 123.24 (C); 127.47 (2× CHAr); 129.15 (C); 129.35 (CHAr); 129.44 (C); 129.75 (2× CHAr); 130.15 (2× CHAr); 130.42 (2× CHAr); 140.41 (C); 153.92 (C); 166.69 (C). ESI (*m*/*z*): calcd for C_26_H_26_Cl_2_N_2_O_2_ 468.1; found 469.14 [M + 1]. IR cm^−1^: 1687 (CO).

*N-(1-(2-Chlorobenzyl)piperidin-4-yl)-2-(2-chlorophenoxy)-N-(3-fluorophenyl) acetamide* (**14d**). Following the general procedure, benzaldehyde derivative **11** (R_3_ = Cl(*o*)) (57.9 mg, 0.4142 mmol); compound **6d** (150 mg, 0.4142 mmol); sodium triacetoxyborohydride (131.6 mg, 0.6213 mmol) and acetic acid (37.3 mg, 0.6213 mmol) in 1,2-dichloroethane (5 mL) was stirred for 24 h at 20 °C to give **14d** (0.11 g, 55%); m.p.: 85 °C; ^1^H-NMR (CDCl_3_, 600 MHz): 1.44 (m, 2H, CH_2_); 1.78 (m, 2H, CH_2_); 2.22 (m, 2H, CH_2_); 2.92 (m, 2H, CH_2_); 3.56 (s, 2H, CH_2_); 4.36 (s, 2H, CH_2_); 4.64 (m, H, CH); 6.73–7.37 (m, 12H aromatic). ^13^C-NMR (150 MHz, CDCl_3_) δ: 30.42 (2× CH_2_); 53.04 (CH); 53.37 (2× CH_2_); 59.17 (CH_2_); 67.74 (CH_2_); 113.95 (2× CHAr); 116.62 (d, *J* = 21 Hz, CHAr); 117.78 (d, *J* = 21 Hz, CHAr); 122.36 (CHAr); 123.25 (C); 126.16 (CHAr); 126.68 (CHAr); 127.66 (CHAr); 128.24 (CHAr); 129.55 (CHAr); 130.59 (CHAr); 130.91 (d, *J* = 10.5 Hz, CHAr); 134.34 (C); 136 (C); 138.44 (d, *J* = 7.5 Hz, C); 153.78 (C); 162.09 (d, *J* = 249 Hz, C); 166.62 (C). ESI (*m*/*z*): calcd for C_26_H_25_Cl_2_FN_2_O_2_ 486.1; found 487.13 [M + 1]; IR cm^−1^: 1688 (CO).

*N-(1-(2.4-Dihydroxybenzyl) piperidin-4-yl)-2-phenoxy-N-phenylacetamide* (**15a**). Following the general procedure for reductive amination using benzaldehyde derivative **11** (R_3_ = OH (*o*), OH (*p*)) (66.86 mg, 0.4838 mmol); compound **6a** (150 mg, 0.4838 mmol); sodium triacetoxyborohydride (153.8 mg, 0.725 mmol) and acetic acid (43 mg, 0.725 mmol) in 1,2-dichloroethane (5 mL) give compound **15a** (0.1233 g, 59%); m.p. 68 °C. ^1^H-NMR (CDCl_3_, 600 MHz): 1.42 (m, 2H, CH_2_); 2.23 (m, 2H, CH_2_); 3.03 (m, 2H, CH_2_); 3.16 (m, 2H, CH_2_); 3.60 (s, 2H, CH_2_); 4.23 (s, 2H, CH_2_); 4.86 (m, H, CH); 5.30 (s, 2× OH); 6.73–7.42 (m, 13HAr). ^13^C-NMR (150 MHz, CDCl_3_) δ: 30.27 (2× CH_2_); 43.66 (2× CH_2_); 51.96 (CH_2_); 52.80 (CH); 67.24 (CH_2_); 114.57 (CHAr); 114.79 (4× CHAr); 121.57 (CHAr); 129.52 (4× CHAr); 129.73 (CHAr); 129.98 (CHAr); 130.09 (CHAr); 136.57 (C); 156.45 (C); 156.66 (C); 157.51 (C); 158.10 (C); 166.78 (C). ESI (*m*/*z*): calcd for C_26_H_28_O_4_ 432.2; found 433.22 [M + 1]; IR cm^−1^: 1661 (CO).

*N-(-1(2-Hydroxy-6-methoxybenzyl)piperidin-4-yl)-2-phenoxy-N-phenylacetamide* (**16a**). Following the general procedure reductive amination using benzaldehyde derivative **11** (R_3_= OMe (*o*), OH (*o*)) (73.55 mg, 0.4838 mmol); compound **6a** (150 mg, 0.4838 mmol); sodium triacetoxyborohydride (153.8 mg, 0.7253 mmol) and acetic acid (43.5 mg, 0.7253 mmol) in 1.2-dichloroethane (5 mL) give the title compound **16a** (0.114 g, 53%); mp 68 °C. ^1^H-NMR (CDCl_3_, 600 MHz): 1.44 (m, 2H, CH_2_); 1.87 (m, 2H, CH_2_); 2.25 (m, 2H, CH_2_); 3.02 (m, 2H, CH_2_); 3.75 (s, 5H, CH_2_, CH_3_); 4.22 (s, 2H, CH_2_); 4.62 (m, H, CH); 6.68–7.38 (m, 13HAr). ^13^C-NMR (150 MHz, CDCl_3_) δ: 30.30 (2× CH_2_); 43.72 (2× CH_2_); 52.45 (CH_2_); 52.62 (CH); 55.61 (CH_3_); 66.68 (CH_2_); 101.61 (CHAr); 109.32 (C); 114.91 (2× CHAr); 121.49 (CHAr); 128.71 (CHAr); 129.49 (2× CHAr); 129.51 (2× CHAr); 130.03 (2× CHAr); 130.06 (2× CHAr); 136.81 (C); 157.71 (C); 158.14 (C); 159.11 (C); 167.54 (C). ESI (*m*/*z*): calcd for C_27_H_30_N_2_O_4_ 446.2; found 447.29 [M + 1]; IR cm^−1^: 1678 (CO).

*N-(1-(Perfluorobenzyl)piperidin-4-yl)-2-phenoxy-N-phenylacetamide* (**17a**). Following the general procedure, benzaldehyde derivative **11** (R_3_ = 5xF (*o*,*m*,*p*)) (94.87 mg; 0.483 mmol); compound **6a** (150 mg, 0.483 mmol); sodium triacetoxyborohydride (153 mg, 0.7258 mmol) and acetic acid (43 mg, 0.7258 mmol) in 1,2-dichloroethane (5 mL) was stirred for 24 h at 20 °C to afford **17a** (0.118 g, 50%); m.p.: 104 °C. ^1^H-NMR (CDCl_3_, 600 MHz): 1.42 (m, 2H, CH_2_); 1.79 (m, 2H, CH_2_); 2.24 (m, 2H, CH_2_); 2.88 (m, 2H, CH_2_); 3.65 (s, 2H, CH_2_); 4.21 (s, 2H, CH_2_); 4.60 (m, H, CH); 6.73–7.42 (m, 10H aromatic). ^13^C-NMR (150 MHz, CDCl_3_) δ: 30.27 (2× CH_2_); 48.64 (CH_2_); 52.18 (2× CH_2_); 52.40 (CH); 66.77 (CH_2_); 114.81 (2× CHAr); 121.46 (CHAr); 129.27 (CHAr); 129.47 (2× CHAr); 129.84 (2× CHAr); 110.16 (C); 130.22 (2× CHAr); 136.56 (C); 136.88 (C); 138.16 (C); 144.42 (2× C); 146.42 (C); 158.15 (C); 167.36 (C). ESI (*m*/*z*): calcd for C_26_H_23_F_5_ N_2_O_2_ 490.2; found 491.17 [M +1]; IR cm^−1^: 1683 (CO).

*N-(3-Fluorophenyl)-N-(1-(perfluorobenzyl)piperidin-4-yl)-2-phenoxyacetamide* (**17b**). Following the general procedure, benzaldehyde derivative **11** (R_3_ = 5xF (*o*,*m*,*p*)) (89.6 mg, 0.457 mmol); compound **6b** (150 mg, 0.457 mmol); sodium triacetoxyborohydride (142.29 mg, 0.685 mmol) and acetic acid (41.16 mg, 0.685 mmol) in 1,2-dichloroethane (5 mL) was stirred for 24 h at 20 °C, to furnish **17b** (0.125 g, 54%); m.p.: 95 °C. ^1^H-NMR (CDCl_3_, 600 MHz): 1.42 (m, 2H, CH_2_); 1.9 (m, 2H, CH_2_); 2.24 (m, 2H, CH_2_); 2.93 (m, 2H, CH_2_); 3.69 (s, 2H, CH_2_); 4.28 (s, 2H, CH_2_); 4.58 (m, H, CH); 6.73–7.42 (m, 9H aromatic). ^13^C-NMR (150 MHz, CDCl_3_) δ: 30.27 (2× CH_2_); 43.66 (2× CH_2_); 51.79 (CH_2_); 52.40 (CH); 67.24 (CH_2_); 113.98 (2× CHAr); 116.90 (d, *J* = 19.5 Hz, CHAr); 117.60 (d, *J* = 21 Hz, CHAr); 122.52 (CHAr); 122.66 (CHAr); 123.18 (C); 125.95 (CHAr); 127.69 (2× CHAr); 130.66 (CHAr); 131.15 (C); 131.29 (d, *J* = 10.5 Hz, CHAr); 137.81 (2× C); 138.26 (2× C); 146.67 (C); 153.70 (C); 162 (d, *J* = 249 Hz, C); 167.77 (C). ESI (*m*/*z*): calcd for C_26_H_25_Cl_2_FN_2_O_2_ 508.2; found 509.11 [M + 1]; IR cm^−1^: 1676 (CO).

*2-(2-Chlorophenoxy)-N-(1-(perfluorobenzyl)piperidin-4-yl)-N-phenylacetamide* (**17c**). Following the general procedure, benzaldehyde derivatives **11** (R_3_ = 5xF (*o*,*m*,*p*)) (85.57 mg; 0.435mmol); compound **6c** (150 mg, 0.435 mmol); sodium triacetoxyborohydride (138 mg, 0.653 mmol) and acetic acid (39.2 mg, 0.653 mmol) in 1,2-dichloroethane (5 mL) was stirred for 24 h at 20 °C. Yield of **17c**: 0.139 g (61%); m.p.: 78 °C; ^1^H-NMR (CDCl_3_, 600 MHz): 1.42 (m, 2H, CH_2_); 1.79 (m, 2H, CH_2_); 2.24 (m, 2H, CH_2_); 2.93 (m, 2H, CH_2_); 3.69 (s, 2H, CH_2,_); 4.28 (s, 2H, CH_2_); 4.58 (m, H, CH); 6.73–7.42 (m, 9H aromatic). ^13^C-NMR (150 MHz, CDCl_3_) δ: 30.27 (2× CH_2_); 48.64 (CH_2_); 52.18 (2× CH_2_); 52.40 (CH); 67.24 (CH_2_); 113.85 (2× CHAr); 122.51 (CHAr); 123.18 (C); 127.61 (2× CHAr); 129.61 (CHAr); 130.11 (CHAr); 130.56 (2× CHAr); 136.27 (C); 136.50 (2× C); 138.47 (2× C); 146.49 (C); 146.31 (C); 153.88 (C); 167.77 (C). ESI (*m*/*z*): calcd for C_26_H_22_ClF_5_ N_2_O_2_ 524.1; found 525.13 [M + 1]; IR cm^−1^: 1683 (CO).

*2-(2-Chlorophenoxy)-N-(3-fluorophenyl)-N-(1-(perfluorobenzyl) piperidin-4-yl) acetamide* (**17d**). Following the general procedure, benzaldehyde derivative **11** (R_3_ = 5xF (*o*, *m*,*p*)) (81.19 mg, 0.4142 mmol); compound **6d** (150 mg, 0.4142 mmol); sodium triacetoxyborohydride (131.6 mg, 0.6213 mmol) and acetic acid (37.3 mg, 0.6213 mmol) in 1,2-dichloroethane (5 mL) was stirred for 24 h at 20 °C to provide **17d** (0.1324 g, 59%); m.p.: 125 °C; ^1^H-NMR (CDCl_3_, 600 MHz): 1.42 (m, 2H, CH_2_); 1.9 (m, 2H, CH_2_); 2.24 (m, 2H, CH_2_); 2.93 (m, 2H, CH_2_); 3.69 (s, 2H, CH_2_); 4.28 (s, 2H, CH_2_); 4.58 (m, H, CH); 6.73–7.42 (m, 8H aromatic). ^13^C-NMR (150 MHz, CDCl_3_) δ: 30.27 (2× CH_2_); 43.66 (2× CH_2_); 51.79 (CH_2_); 52.40 (CH); 67.24 (CH_2_); 113.98 (CHAr); 116.90 (d, *J* = 19.5 Hz, CHAr); 117.60 (d, *J* = 21 Hz, CHAr); 122.52 (CHAr); 122.66 (C); 123.18 (C); 125.95 (CHAr); 127.69 (CHAr); 130.66 (CHAr); 131.15 (C); 131.29 (d, *J* = 10.5 Hz, CHAr); 137.81 (2× C); 138.26 (2× C); 146.67 (C); 153.70 (C); 162 (d, *J* = 249 Hz, C); 167.77 (C). ESI (*m*/*z*): calcd for C_26_H_22_ClF_5_ N_2_O_2_ 542.1; found 543.13 [M + 1]; IR cm^−1^: 1695 (CO).

### 3.3. Biological Assays

#### 3.3.1. Antiplasmodial Assay

The antimalarial activity of extracts/compounds was evaluated against *P. falciparum* 3D7 and *P. falciparum* W2 strains, using the fluorescence-based SYBR Green I assay approach in 96-well microplates as described by Smilkstein et al. [34] with some modifications. Positive control wells for each assay contained no inhibitor while negative controls contained Chloroquine (CQ). The CQ molecule was provided from World Wide Antimalarial Resistance Network (wwarn Network). Experiments were run in duplicate with both test and control drugs employed at varying concentrations. Stock solutions (extracts) were prepared in dimethyl-sulfoxide (DMSO) and diluted with culture medium to give a maximum DMSO concentration of 0.5% in a final well volume of 200 μL containing 1% parasitemia and 2.5% haematocrit. Extracts and negative control (chloroquine (CQ)) were prepared by two-fold dilution, in a dose-titration range of 0.098–100 µg/mL, to obtain 11 concentrations each, in duplicate. The concentrations used for CQ were between 0.5 and 1000 nM. After 48 h incubation, the plates were subjected to 3 freeze thaw cycles to achieve complete hemolysis. The parasite lysis suspension was diluted 1:5 in SYBR Green I lysis buffer (10 mM NaCl, 1 mM Tris HCl pH 8, 2.5 mM EDTA pH 8, 0.05% SDS, 0,01 mg/mL proteinase K and 10X SYBR Green I). Incorporation of SYBR Green I in parasite DNA amplification was measured using the Master epRealplex cycler^®^ (Eppendorf, Montesson, France) according the following program to increase the SYBR green incorporation: 90 °C for 1 min, decrease in temperature from 90 °C to 10 °C for 5 min with reading the fluorescence 10 °C for 1 min and a new reading at 10 °C for 2 min. The IC_50_ was calculated by nonlinear regression using icestimator website 1.2 version: http://www.antimalarial-icestimator.net/MethodIntro.htm.

#### 3.3.2. Cytotoxicity on HUVEC

HUVEC cells were cultured in Gibco™ RPMI 1640 medium (Life Technologies, Saint-Aubin, France) complemented with 10% Fetal Bovine Serum and 1 mM l-glutamine (Sigma-Aldrich, Lesquin, France) and incubated in 5% CO_2_ at 37 °C. The cytotoxicity of extracts was evaluated using the SYBR Green I assay as previously described. HUVEC were seeded in a 96-well plate at 100,000 cells/well and incubated for 24 h to adhere. After discarding the old medium, the cells were incubated in the medium containing eight concentrations (0.78–100 μg/mL) of each extract in duplicate. After 48 h incubation, cells were visualized using an inverted microscope to check their morphology or the cell viability. The medium was subsequently removed and replaced by lysis buffer without SYBR Green I and the plates were subjected to 3 freeze-thaw cycles. The cell lysis suspension was diluted 1:2 in SYBR Green I lysis buffer. The incorporation of SYBR Green I in cell DNA and the IC_50_ analysis were obtained as previously.

## 4. Conclusions

In this study, we have prepared a small library of new nitrogen heterocycles displaying piperidine scaffolds using a flexible synthetic approach. Eighteen new derivatives were prepared in good yield. The antimalarial activity of these compounds has been described. The compounds were tested against *P*. *falciparum* 3D7 strains and W2. The best result is observed with the compounds **13b** against the 3D7 strain and **12a** against the W2 one with a selectivity index greater than chloroquine. We observed that modification with different R groups, for example compound **12a** (R_1_ = R_2_ = R_3_ = H) in **13b** (R_1_ = F, R_2_ = H, R_3_ = Br) significantly modulated the activity of the tested molecules. These molecules could be further optimized to provide good malaria drug candidates.

## Figures and Tables

**Table 1:** The products of the synthesis of **6** recorded.

Compounds	R_1_	R_2_	Overall Yields %
**6a**	H	H	35
**6b**	F	H	34
**6c**	H	Cl	64
**6d**	F	Cl	33

**Table 2:** The target compounds **A.**

Compounds	R_1_	R_2_	R_3_	Time (h)	Overall Yields %
**12a**	H	H	H	24	18
**12b**	F	H	H	24	18
**12c**	H	Cl	H	24	32
**12d**	F	Cl	H	24	18
**13a**	H	H	Br (*o*)	24	21
**13b**	F	H	Br (*o*)	24	19
**13c**	H	Cl	Br (*o*)	24	35
**13d**	F	Cl	Br (*o*)	24	20
**14a**	H	H	Cl (*o*)	24	18
**14c**	H	Cl	Cl (*o*)	24	41
**14d**	F	Cl	Cl (*o*)	24	18
**15a**	H	H	OH (*o*), OH (*p*)	24	24
**16a**	H	H	OH (*o*), OMe (*o*)	24	18
**17a**	H	H	5xF (*o*,*m*,*p*)	24	17
**17b**	F	H	5xF (*o*,*m*,*p*)	24	18
**17c**	H	Cl	5xF (*o*,*m*,*p*)	24	39
**17d**	F	Cl	5xF (*o*,*m*,*p*)	24	20

**Table 3:** The antimalarial activity of compounds derivatives **6**.

	*Plasmodium falciparum* 3D7 Strain	*Plasmodium falciparum* W2 Strain	HUVEC Cells	Selectivity Index (3D7)	Selectivity Index (W2)
Compounds	IC50 ± SD (nM)	IC_50_ ± SD (nM)	CC_50_ nM ± SD	=CC_50_/IC_50_	=CC_50_/IC_50_
**6a**	34.46 ± 9.25	61.37 ± 11.12	nd	nd	nd
**6b**	17.42 ± 7.7	30.35 ± 6.09	>100	>17.5	>10.8
**6c**	>100	>100	/	/	/
**6d**	>100	>100	/	/	/
**CQ**	22.38 ± 3.24	134.12 ± 32.29	37.56 ± 1.24	1.7	0.3

**Table 4:** The antimalarial activity of the target compounds.

	*Plasmodium falciparum* 3D7 Strain	*Plasmodium falciparum* W2 Strain	HUVEC Cells	Selectivity Index (3D7)	Selectivity Index (W2)
Compounds	IC_50_ ± SD (nM)	IC_50_ ± SD (nM)	CC_50_ nM ± SD	=CC_50_/IC_50_	=CC_50_/IC_50_
**10**	25.37 ± 2.88	42.14 ± 6.73	>100	>8.9	>5.3
**12a**	36.9 ± 6.59	11.06 ± 4.82	100 ± 0.008	2.85	5.7
**12b**	34.45 ± 7.36	38.95 ± 3.66	>100	>6.9	>6.1
**12c**	>100	>100	/	/	/
**12d**	13.64 ± 2.47	166.87 ± 9.64	nd	nd	nd
**13a**	>100	>100	/	/	/
**13b**	4.19 ± 1.12	13.30 ± 2.01	112 ± 0.008	26.7	8.4
**13c**	44.17 ± 3.9	28.57 ± 1.91	>100	>4.4	>6.8
**13d**	>100	>100	/	/	/
**14a**	20.72 ± 7.69	32.33 ± 9.97	52 ± 0.004	2.5	1.6
**14c**	50.33 ± 3.8	18.97 ± 7.30	>100	>4.2	>11.3
**14d**	14.85 ± 4.48	23.45 ± 4.66	100 ± 0.005	6.83	4.3
**15a**	˃100	˃100	/	/	/
**16a**	˃100	˃100	/	/	/
**17a**	37.63 ± 7.85	47.84 ± 5.83	nd	nd	nd
**17b**	˃100	˃100	/	/	/
**17c**	14.65 ±2.55	36.88 ± 2.99561	nd	nd	nd
**17d**	˃100	˃100	/	/	/
**CQ**	22.38 ± 3.24	134.12 ± 32.29	37.56 ± 1.24	1.7	0.3

## References

[1] World Health Organization (2017). World Malaria Report.

[2] Noedl H. (2013). The need for new antimalarial drugs less prone to resistance. Curr. Pharm. Des..

[3] Njuguna N., Ongarora D.S.B., Chibale K. (2012). Artemisin derivatives: A patent review (2006-present). Expert. Opin. Ther. Pat..

[4] Ongarora D.S.B., Strydom N., Wicht K., Njoroge M., Wiesner L., Egan T.J., Wittlin S., Jurva U., Masimirembwa C.M., Chibale K. (2015). Antimalarial benzoheterocyclic 4-aminoquinolines: Structure-activity relationship, in vivo evaluation, mechanistic and bioactivation studies. Bioorg. Med. Chem..

[5] Toshio H., Fumihiro I. (2000). Shin-Ichi, Y. Direct Arylation of 2-Pyridones; Photostimulated SRN1 Reaction between Cesium Phenoxides and Chloro-2-pyridones. Heterocycles.

[6] Kohnen-Johannsen K.L., Kayser O. (2019). Tropane Alkaloids: Chemistry, Pharmacology, Biosynthesis and Production. Molecules.

[7] Huang J.P., Fang C., Ma X., Wang L., Yang J., Luo J., Yan Y., Zhang Y., Huang S.X. (2019). Tropane alkaloids biosynthesis involves an unusual type III polyketide synthase and non-enzymatic condensation. Nat. Commun..

[8] Santos A.S., Lukens K.A., Coelho L., Noguero F., Wirth D.F., Mazitschek R., Moreira R., Paulo A. (2015). Exploring the 3-piperidin-4-yl-1H-indole scaffold as a novel antimalarial chemotype. Eur. J. Med. Chem..

[9] Ishiguro Y., Kubota T., Ishiuchi K., Fromont J., Kobayashi J. (2009). A novel piperidine alkaloid from an Okinawan marine sponge Plakortis sp.. Tetrahedron Lett..

[10] Kubizna P., Spanik I., Kozisek J., Szolcsanyi P. (2010). Synthesis of 2,6-disubstituted piperidine alkaloids from ladybird beetles Calvia 10-guttata and Calvia 14-guttata. Tetrahedron.

[11] Gassama A., Ernenwein C., Hoffmann N. (2009). Photochemical Key Steps in the Synthesis of Surfactants; from Furfural-Derived Intermediates. ChemSusChem.

[12] Weis R., Schweiger K., Faist J., Rajkovic E., Kungl A.J., Fabian W.M.F., Schunack W., Seebacher W. (2008). Antimycobacterial and H1-antihistaminic activity of 2-substituted piperidine derivatives. Bioorg. Med. Chem..

[13] Dambuza N.S., Smith P., Evans A., Norman J., Taylor D., Andayi A., Egan T., Chibale K., Wiesner L. (2015). Antiplasmodial activity, in vivo pharmacokinetics and anti-malarial efficacy evaluation of hydroxypyridinone hybrids in a mouse model. Malar. J..

[14] Sun H., Scott O.D. (2011). Metabolism of 4-Aminopiperidine Drugs by Cytochrome P450s: Molecular and Quantum Mechanical Insights into Drug Design. Med. Chem. Lett..

[15] Heringa M. (2003). Review on raloxifene: Profile of a selective estrogen receptor modulator. Int. J. Clin. Pharmacol. Ther..

[16] Vogel V., Constantino J.P., Wickerman L. (2006). Effects of tamoxifen vs raloxifene on the risk of developing invasive breast cancer and other disease outcomes: The NSABP Study of Tamoxifen and Raloxifene. JAMA.

[17] Wei X., Nieves K., Rodríguez A.D. (2010). Neopetrosiamine A, biologically active bis-piperidine alkaloid from the Caribbean Sea sponge *Neopetrosia proxima*. Bioorg. Med. Chem. Lett..

[18] Varty G.B., Cohen-Williams M.E., Hunter J.C. (2003). The antidepressant-like effects of neurokinin NK1 receptor antagonists in a gerbil tail suspension test. Behav. Pharmacol..

[19] Varty G.B., Cohen-Williams M.E., Morgan C.A. (2002). The gerbil elevated plus-maze II: Anxiolytic-like effects of selective neurokinin NK1 receptor antagonists. Neuropsychopharmacology.

[20] Watanabe Y., Asai H., Ishii T., Kiuchi S., Okamoto M., Taniguchi H., Nagasaki M., Saito A. (2008). Pharmacological characterization of T-2328, 2-fluoro-4′-methoxy-3′-[[[(2*S*,3*S*)-2-phenylpiperidinyl]-amino]methyl]-[1,1′-biphenyl]-4-carbonitrile dihydrochloride, as a brain-penetrating antagonist of tachykinin NK1 receptor. J. Pharm. Sci..

[21] Watson P.S., Jiang B., Scott B. (2000). A Diastereoselective Synthesis of 2.4-Disubstituted Piperidines: Scaffolds for Drug Discovery. Org. Lett..

[22] Padmanilayam M., Scorneaux B., Dong Y., Chollet J., Matile H., Charman S.A., Creek D.J., Charman W.N., Tomas S.T., Scheurer C. (2006). Antimalarial activity of N-alkyl amine, carboxamide, sulfonamide and urea derivatives of a dispiro-1,2,4-trioxolane piperidine. Bioorg. Med. Chem. Lett..

[23] Meyers M.J., Anderson E.J., McNitt S.A., Krenning T.M., Singh M., Xu J., Zeng W., Qin L., Xu W., Zhao S. (2015). Evaluation of spiropipéridine hydantoins as a novel class of antimalarial agent. Bioorg. Med. Chem..

[24] Misra M., Pandey S.K., Pandey V.P., Pandey J., Tripathi R., Tripathi R.P. (2009). Organocatalyzed highly atom economic one pot synthesis of tetrehydropyridines as antimalarials. Bioorg. Med. Chem..

[25] Sabbani S., Stocks P.A., Ellis G.L., Davies J., Hedenstrom E., Ward S.A. (2008). O’Neill, P.M. Piperidine dispiro-1,2,4-trioxane analogues. Bioorg. Med. Chem. Lett..

[26] Kikuchi H., Tasaka H., Hirai S., Takaya Y., Iwabuchi Y., Ooi H., Hatakeyama S., Kim H.S., Wataya Y., Oshima Y. (2002). Potent Antimalarial Febrifugine Analogues Against the *Plasmodium* Malaria Parasite. J. Med. Chem..

[27] Gassama A., Diatta A. (2015). Synthesis of N-Substituted piperidines from piperidone. J. Soc. Ouest-Afr. Chim..

[28] Amed F., Magid A., Cynthia A., Kenneth M., Carson G. (1990). Reductive amination of aldehydes and ketones by using sodium triacetoxyborohydride. Tetrahedron Lett..

[29] Borch R.F., Bernstein M.D., Durst H.D. (1971). Cyanohydridoboration anion as a selective reducing agent. J. Am. Chem. Soc..

[30] Morandi G., Kebir N., Campistron I., Gohier F., Laguerre A., Pilard J.F. (2007). Direct selective reductive amination of carbonyl telechelic oligoisoprenes: Elaboration of promising tri and tetrafunctionalized oligoisooprene intermediates. Tetrahedron Lett..

[31] Khan S.N., Bae S.Y., Kim H.S. (2005). A highly stereoselective reductive amination of 3-ketosteroid with amines: Improved synthesis of 3α-aminosteroids. Tetrahedron Lett..

[32] Adachi K., Tsuru E., Banjyo S.E.K., Yamashita T. (1998). Selective BH 3-Reduction of Amide Carbonyl Groups of Lithium Salts of N-t-Butoxycarbonyl (S)-O-Benzyl Tyrosyl (*S*)-Proline and N,N’-Ethylene-Bridged Dipeptides. Synthesis.

[33] Beamson G., Papworth A.J., Philipps C., Smith A.M., Whyman R. (2010). Selective hydrogenation of amides using Rh/Mo catalysts. J. Cat..

[34] Komlaga G., Genta-Jouve G., Cojean S., Dickson R.T., Mensah M.L.K., Loiseau P.M., Champy P., Beniddir M.A. (2017). Antiplasmodial Securinega alkaloids from *Phyllanthus fraternus*: Discovery of natural (+)− allonorsecurinine. Tetrahedron Lett..

